# Action and Contribution of the Iliopsoas and Rectus Femoris as Hip Flexor Agonists Examined with Anatomical Analysis

**DOI:** 10.14789/jmj.JMJ22-0009-OA

**Published:** 2022-06-20

**Authors:** TOSHIMASA KUMAZAKI, TOMIHISA TAKAHASHI, TAKASHI NAKANO, TATSUO SAKAI

**Affiliations:** 1 Department of Health and Sports Management, Osaka University of Health and Sport Sciences, Osaka, Japan; 1 Department of Health and Sports Management, Osaka University of Health and Sport Sciences, Osaka, Japan; 2 Department of Anatomy, Nihon University School of Dentistry, Tokyo, Japan; 2 Department of Anatomy, Nihon University School of Dentistry, Tokyo, Japan; 3 Department of Anatomy, School of Medicine, Aichi Medical University, Aichi, Japan; 3 Department of Anatomy, School of Medicine, Aichi Medical University, Aichi, Japan; 4 Department of Physical Therapy, Faculty of Health Science, Juntendo University, Tokyo, Japan; 4 Department of Physical Therapy, Faculty of Health Science, Juntendo University, Tokyo, Japan; 5 Department of Anatomy and Life Structure, Juntendo University Graduate School of Medicine, Tokyo, Japan; 5 Department of Anatomy and Life Structure, Juntendo University Graduate School of Medicine, Tokyo, Japan

**Keywords:** hip joint, iliopsoas, rectus femoris, physiological cross-sectional area, torque

## Abstract

**Objectives:**

To evaluate the difference in action between the iliopsoas and rectus femoris muscles in hip flexion by estimating the relative contribution to the maximal hip flexion torque and relative rotation speed.

**Materials:**

We examined 22 lower limbs of 10 male and 12 female formaldehyde-fixed adult Japanese cadavers.

**Methods:**

Using morphometric data from cadaver dissections, we calculated the moment arm length and physiological cross-sectional area for each muscle. We considered moment arm length and physiological cross-sectional area as indices of the maximal torque and compared them among the muscles at various hip joint angles. To evaluate the relative rotation speed, we calculated the increase of the hip joint angle for a 1% reduction of the muscle fiber length in each muscle.

**Results:**

The rectus femoris contributed approximately 2/3 to the flexion torque in mild flexion up to 60°, whereas the iliopsoas contribution increased sharply beyond 60°. The relative iliopsoas rotation speed was 2.5- to 3-times higher than that of the rectus femoris in mild flexion up to 60° under the specific condition that each muscle had the same muscle contraction speed.

**Conclusions:**

We found that the iliopsoas served as a rapid flexor, while the rectus femoris was a powerful flexor.

## Introduction

Among the activities of daily living, standing up, as well as walking, constitute the most critical and fundamental exercises^1-2)^. In these exercises, flexion and extension of the hip and knee joints are essential and critical motor elements^3)^. The extension of the hip and knee joints plays an important role during chair-based standing and sitting. Various studies have analyzed the biomechanics of these extension activities using force plates^1, 4)^, motion analysis^2, 4, 5)^, and electromyography (EMG)^1, 2, 4, 6)^. The walking and running exercises involve flexion of the hip and knee joints. Based on their observations of larger hip flexor muscles in top sprinter athletes, Ema et al^7)^. reported that hip flexion could play an important role in track and field sprinting. Biomechanical analysis results of these flexion activities have been reported in studies employing EMG of the iliopsoas and rectus femoris^8)^ or combining EMG and hip flexion torque measurement^9)^.

Joint movements are exerted in general by multiple muscles. During knee extension, the four muscles of the quadriceps femoris play the role of the main agonist. During hip extension, the gluteus maximus and three muscles of the hamstrings play the role of main agonists, and the adductor maximus acts as a synergist. During hip flexion, the iliopsoas and rectus femoris are the main agonists, and the sartorius and adductor longus act as synergists. Dynamometers measure the total torque produced by multiple agonists and synergists. However, it cannot discriminate the muscle strength of individual muscles. The EMG estimates the change of activities in individual muscles during joint movements. However, it cannot predict the relative muscle strength among the agonists and synergists. The EMG activities of the superficially located rectus femoris have been reported in many studies^7, 10-17)^, and those of the deeply located iliopsoas have also been reported in some studies^8, 18-23)^.

The morphometric parameters of the skeletal muscles, such as the muscle fiber length (FL) and physiological cross-sectional area (PCSA), are well known to affect the muscle function, including the contraction speed, maximum muscle strength, and the effective contractible range^24, 25)^. Indeed, the agonists and synergists of the hip and knee joints reportedly have different architectural parameters and functional characteristics^26)^, suggesting that the multiple flexors and extensors of the hip and knee joints have varying contributions to the torque and speed during joint movement. However, the contribution of the individual muscles was not hitherto revealed by the previous studies employing dynamometers, EMG, and other physiological methods.

The iliopsoas (psoas major and iliacus), rectus femoris, sartorius, and adductor longus are known as the hip flexor muscles. The iliopsoas and rectus femoris are considered agonist muscles among the hip flexor muscles as they have greater PCSA values^26)^. In comparison, the sartorius and adductor longus have much smaller PCSA values, and their force vector is deviated from the sagittal plane, so they are thought to have only minor contributions compared with the iliopsoas and rectus femoris. In the present study, we determined the relative contribution of the iliopsoas and rectus femoris to the maximal hip flexion torque based on morphological methods by estimating PCSA and moment arm length (MAL) values in the sagittal plane. Additionally, based on the morphometric data of the skeletal anatomical specimens, we estimated the flexion speed of individual muscles by calculating the degree of flexion produced by 1% of shortening of the hip flexor muscles. The data provided by the present methods were not relevant to estimate the actual torque and speed of the joint movements but revealed the maximal and relative contributions of the individual muscles on a theoretical basis.

## Materials and Methods

### Materials

We used 22 lower limbs of 10 male and 12 female formaldehyde-fixed adult Japanese cadavers with no apparent degeneration in the hip and knee joints that were used in the dissection course at the Nihon University School of Dentistry during the 2014 and 2016 academic years. For morphometry and estimation of the PCSA of the iliopsoas and rectus femoris, 12 specimens from 6 male and 6 female cadavers (age at death, 78.6 ± 10.6 years) were used, and for measurement of the MAL, 10 specimens from 4 male and 6 female cadavers (age at death, 79.6 ± 6.8 years) were used. The body donors gave written informed consent for the donation of their tissues for research and teaching purposes. The study was carried out following all relevant guidelines and regulations and was approved by the ethical committee of the Nihon University School of Dentistry (EP14D009, EP16D017).

### Procedures/protocol

Morphometry and calculation of PCSA in isolated muscle specimens

In the 12 lower limbs, the iliopsoas and rectus femoris were dissected out to prepare the isolated muscle specimens as stated previously^27, 28)^.

In the isolated muscle specimens, the mass (M), FL, and pennation angle (θ) were measured. The pennation angles were measured with a protractor at the distal myotendinous junction as the angular deviation between the muscle fibers and tendon^28)^. The FL and pennation angles were measured and averaged in three places (superficial, intermediate, and deep portions) on the iliopsoas and in three places (on the left, right, and distal sides of the origin tendon) on the rectus femoris.

Based on these data, the PCSA was calculated using the following formula, with the density of mammalian muscles represented by ρ (ρ=1.056 g/cm^3^)^29)^:

PCSA (cm^2^) = M (g) × cos θ/ρ (g/cm^3^) × FL (cm)^30-32)^.

Measurement of muscle MAL in skeletal specimens

The 10 lower limbs were dissected, and the locations of the origin and insertion of the iliopsoas (the psoas major and iliacus) and rectus femoris were marked on the skeleton. Then, the muscles were removed to prepare the skeleton specimens. The diameter of the femoral head was measured after opening the hip joint and removing the iliofemoral and pubofemoral ligaments covering the head. Three-dimensional reference values were obtained with two reflective markers for the origin and insertion of the muscle on the skeleton, and four reflective markers on the surface of the femoral head for the joint center of the hip joint.

A) Measurement of three-dimensional reference values in the hip extension position.

The MAL in the hip extension position was calculated from the three-dimensional reference values of the reflective markers attached to the skeleton that were measured with four infrared cameras and an optical motion capture system (NaturalPoint, Inc., Corvallis, OR, USA). The origin of the psoas major was marked on the T12 for the superficial head and on the costal process of L1 for the deep head, and the insertion was marked on the lesser trochanter of the femur^33)^. The origin and insertion of the iliacus were marked on top of the iliac crest and the lesser trochanter, respectively. The origin and insertion of the rectus femoris were marked on the ridge of the anterior inferior iliac spine and the tibial tuberosity, respectively. The center of the hip joint was determined from the four markers attached to the femoral head surface, and the offset distance in the direction of the ball center representing the radius was determined by direct measurement.

B) Estimation of MAL under different hip joint angles in the sagittal plane.

The MAL of the muscles was calculated from the three-dimensional reference values of the reflection markers projected on the sagittal plane as the distance between the center of the hip joint and the vector of the muscle force by Skycom software (Optitrack Japan, Tokyo, Japan). The iliopsoas did not take a straight course between the origin and the insertion in the hip extension positions in various degrees up to 60°, but a curved course around the femoral head, so that the MAL was represented by the radius of the femoral head.

The psoas major (PM) had two heads whose origins were represented by the T12 vertebral body for the superficial head (PM-I) and by the L1 transverse process for the deep head (PM-II). The iliacus (IL) had a broad origin region on the iliac fossa, and the origins were represented by four points on the iliac crest including the anterior superior iliac spine (IL-I), the midpoint between the former and the apex of the iliac crest (IL-II), the apex of the iliac crest (IL-III), and the posterior superior iliac spine (IL-IV). The MAL was represented by the radius of the femoral head for IL-I in joint angles up to 10°, IL-II up to 30°, PM-I up to 50°, and IL-III, IL-IV, and PM-II up to 60°, and by the length of the vertical line from the hip joint center to the vector from the origins to the insertion (lesser trochanter of the femur) in larger joint angles. We employed the mean value of the MAL of PM-I and PM-II for the PM, and of IL-I to IL-IV for the IL.

**Figure 1:**
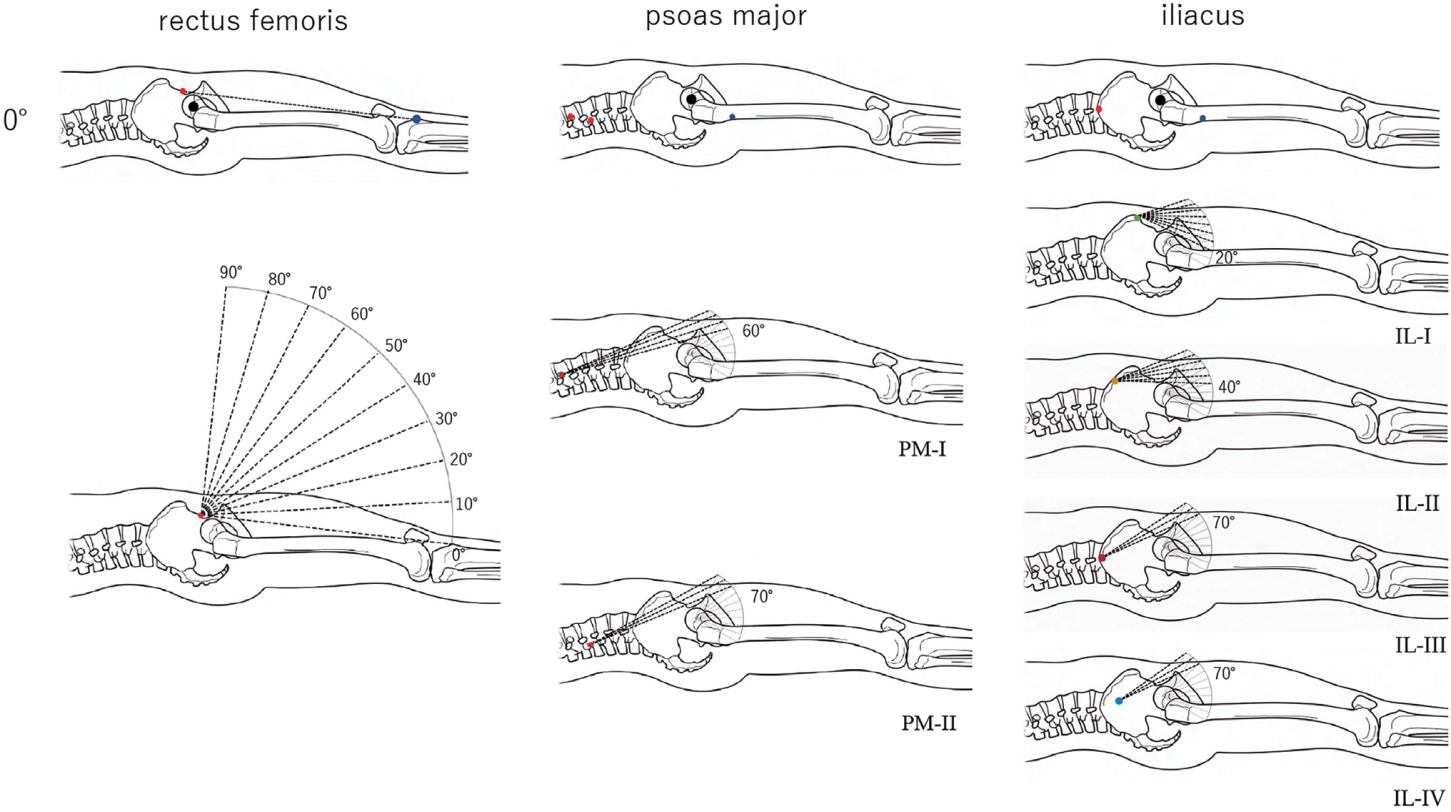
Measurement of moment arm length (MAL) of the three muscles under different hip joint angles. For the rectus femoris, the MAL was represented by the length of the vertical line from the hip joint center (black dot) to the vector from the origin (red dot) to the insertion (blue dot) in all angle regions. For the psoas major (PM), PM had two heads, and the origins were represented by the T12 vertebral body for the superficial head (PM-I) and by the L1 transverse process for the deep head (PM-II). The MAL was represented by the radius of the femoral head for PM-I up to 50° and for PM-II up to 60°, and by the length of the vertical line from the hip joint center to the vector from the origins (red dot) to the insertion in larger joint angles. The iliacus (IL) had a broad origin region on the iliac fossa, which were represented by 4 points (green dot, yellow dot, red dot, and light blue dot) on the iliac crest including the anterior superior iliac spine (IL-I), the midpoint between the former and the apex of the iliac crest (IL-II), the apex of the iliac crest (IL-III), and the posterior superior iliac spine (IL-IV). The MAL was represented by the radius of the femoral head for IL-I in joint angles up to 10°, for IL-II up to 30°, for IL-III and IL-IV up to 60°, and by the length of the vertical line from the hip joint center to the vector from the origins to the insertion in larger joint angles.

For the rectus femoris, the MAL was represented by the length of the vertical line from the hip joint center to the vector from the origin (inferior anterior iliac spine) to the insertion (tibial tuberosity) in all of the angle regions (Figure 1).

Estimation of the relative contribution to the maximal hip flexion torque

To estimate the relative contribution of the individual flexors to the maximal hip flexion torque, we calculated an indicator of maximal hip flexion torque produced by the PM, IL, and rectus femoris as the product of the PCSA and the MAL. The indicators were calculated for individual muscles, and the relative contribution was calculated as the ratios of indicators to the total hip flexion torque for the three muscles at various hip joint angles from extension to flexion (0°-90°).

Estimation of the relative rotation speed by contraction of the flexors

As an indicator of rotation speed by contraction of the PM, IL, and rectus femoris, the change of flexion angle (Δα) due to the 1% shortening of the muscle fiber (ΔFL) was calculated from the muscle FL, pennation angle, and MAL (R) by the following equations under the specific condition that each muscle has the same muscle contraction speed:

ΔFL = FL × 0.01, and

Δα = arctan (ΔFL × cos θ / R) (equation 1).

The equations were derived from the following geometrical reasoning. The shortening of the muscle length (ΔML) and the ΔFL were correlated with the following equation:

ΔML = ΔFL × cos θ

The ΔML was thought to flex the joint by the angle change Δα with the equation:

ΔML = R × tan Δα

The two equations gave an equation from which equation (1) was derived:

tan Δα = ΔFL × cos θ / R

### Data analyses

The morphometric and calculated values including PCSA were analyzed by one-way analysis of variance and multiple comparison tests (Tukey’s honest significant difference test, Bonferroni). All values are presented as mean ± standard deviation. Statistical analyses were conducted using the IBM SPSS version 23 (IBM Corporation, Armonk, NY, USA), and the significance level for all tests was set at P<0.05.

## Results

### Anatomy of the iliopsoas and rectus femoris

The PM, with two heads arising from two different origins, originated as the superficial head from the intervertebral discs between T12 and L4 and the adjacent rims of the vertebral bodies, and as the underlying head from the costal process of L1-L5 and the twelfth rib, and converged to form the insertion tendon ending on the lesser trochanter of the femur. The IL, originating from the iliac fossa and the anterior inferior iliac spine, terminated mainly via the insertion tendon and partly directly without the tendon on the lesser trochanter. The psoas minor, originating from the vertebral body of T12 and L1 and inserting onto the iliac fascia at the iliopubic ramus, contributed little to the flexion of the hip joint. The rectus femoris originated at the anterior inferior iliac spine and the upper part of the acetabulum and inserted onto the tibial tuberosity through the patella.

**Figure 2:**
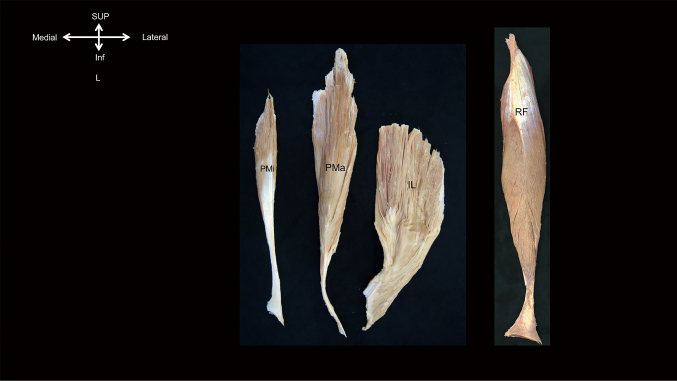
Photographs of isolated muscle specimens of the iliopsoas and rectus femoris in the left-side and superficial views. PMi, psoas minor; PMa, psoas major; IL, iliacus; RF, rectus femoris

The PCSA of the iliopsoas (PM, IL) and the rectus femoris was estimated from the morphometric data obtained from the isolated muscle specimens (Figure 2). The muscle FL of the iliopsoas (PM: 13.76 cm, IL: 11.10 cm) was longer than that of the rectus femoris (8.04 cm) (P<0.01). The PCSA of the rectus femoris (10.88 cm^2^) was larger than that of the PM (5.45 cm^2^) (P<0.05). The PCSA of the iliopsoas was comparable with that of the rectus femoris (Table 1).

**Table 1:** Parameters of the mechanical architecture of the iliopsoas and rectus femoris

	Muscle mass(g)	θ(°)	FL(cm)	PCSA(cm^2^)
Psoas major	81.4±47.4	6.3±2.1	13.76±1.19**	5.45±2.85*
Iliacus	84.1±47.1	7.9±2.6	11.10±1.74**	6.91±2.98
Rectus femoris	90.4±52.8	6.5±2.0	8.04±1.23**	10.88±6.00*

θ, pennation angle; FL, muscle fiber length; PCSA, physiological cross-sectional area **P<0.01, *P<0.05

### MAL under different hip joint angles in the sagittal plane

**Figure 3:**
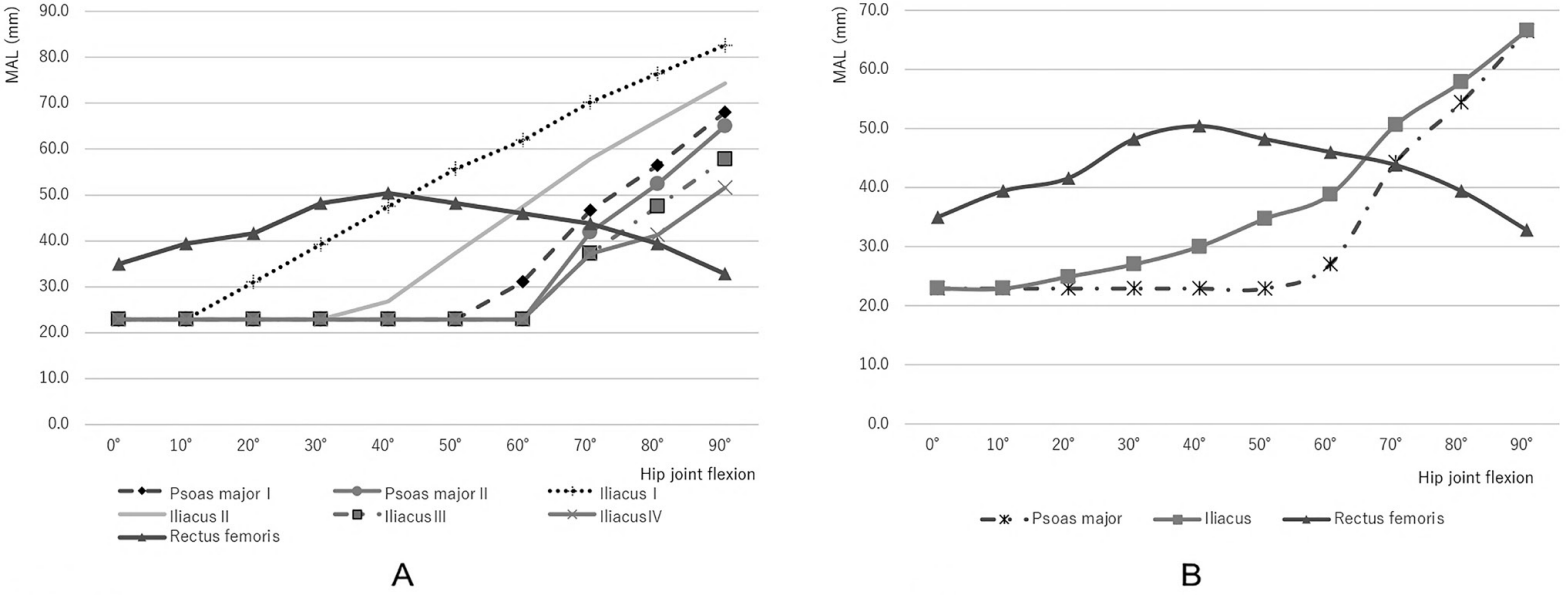
Moment arm length (MAL) under different hip joint angles on the sagittal plane. A. MAL of the iliopsoas (PM-I・II, IL- I -Ⅳ) and rectus femoris estimated in different hip joint angles. The MAL of the rectus femoris increased gradually in mild flexions to reach a maximal value at 40° and decreased gradually in deep flexions up to 90°. The MAL of PM-I was slightly larger than that of PM-II between 60° and 90° and exhibited a similar pattern of change during hip flexions. The MAL of IL-I and IL-II almost remained constant up to 10° and 30°, and thereafter in deep flexions, increased steeply up to 90°, exceeding that of the rectus femoris at the flexion between 40° and 60°. The MAL of IL-Ⅲ and IL-Ⅳ remained constant up to 50° and 60°, increased steeply up to 90°, exceeding that of the rectus femoris at the flexion between 70° and 80°. B. MAL of the iliopsoas and rectus femoris estimated in different hip joint angles. The MAL of the rectus femoris was much larger than that of the iliopsoas in the hip extension position (0°). It increased gradually in mild flexion up to 40° and then decreased gradually. The MAL of the psoas major and iliacus remained unchanged in moderate flexions up to10° and 60°, and thereafter increased in deep flexions up to 90°, exceeding that of the rectus femoris at the flexion between 60° and 70°.

In the hip extension position (0°), the MAL of the rectus femoris (35.0 ± 4.0 mm) was larger than that of PM and IL (22.9 ± 1.9 mm). When the hip joint was flexed, the MAL of the rectus femoris increased gradually in mild flexions to achieve a maximal value at 40° (50.4 mm) and decreased gradually in deep flexions up to 90°. The MAL of PM-I was slightly larger than that of PM-II between 60° and 90° and exhibited a similar pattern of change during hip flexions. The MAL of IL-I and IL-II remained mostly constant up to 10° and 30°, and thereafter in deep flexions, increased steeply up to 90°, exceeding that of the rectus femoris at the flexion between 40° and 60°. The MAL of IL-Ⅲ and IL-Ⅳ remained constant up to 50° and 60°, and increased steeply up to 90°, exceeding that of the rectus femoris at the flexion between 70° and 80° (Figure 3-A).

The MAL of the PM remained unchanged in moderate flexions up to 50° and thereafter increased steeply in deep flexions, exceeding that of the rectus femoris at the flexion between 60° and 70°. Conversely, the MAL of the IL increased gradually in deep flexions, exceeding that of the rectus femoris at the flexion between 60° and 70° (Figure 3-B).

### Relative contribution of individual hip flexors to the maximal hip flexion torque

**Figure 4:**
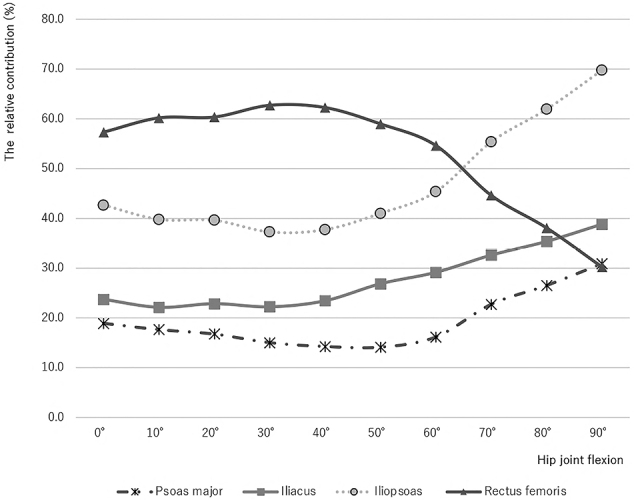
The relative contribution of the iliopsoas and rectus femoris to the maximal hip flexion torque. The relative contribution of the rectus femoris to the maximal hip flexion torque was greater than that of the iliopsoas in mild flexion up to 60°, and its relative contribution decreased steeply in deep flexion. Moreover, the relative contribution of the iliopsoas to the torque increased sharply in the deepest flexions at 80° and 90°.

The relative contribution of the rectus femoris in the hip extension position at 0° (57.3%) exceeded that of the iliopsoas and increased gradually in flexions to an almost maximal value at 20°-40° (60.4%-62.3%); thereafter, it decreased drastically in deep flexions up to 90°, falling behind that of the iliopsoas at a flexion of approximately 60°-70°. The relative contribution of the IL in the hip extension position at 0º remained unchanged in moderate flexions up to 40° and thereafter increased gradually in deep flexions up to 90°, exceeding that of the rectus femoris at the flexion between 80° and 90°. The relative contribution of the PM decreased gradually in flexions up to 50° and thereafter increased in deep flexions up to 90° to become the same level as the rectus femoris in the hip extension position at 90° (Figure 4).

### Relative rotation speed by contraction of the flexors

**Figure 5:**
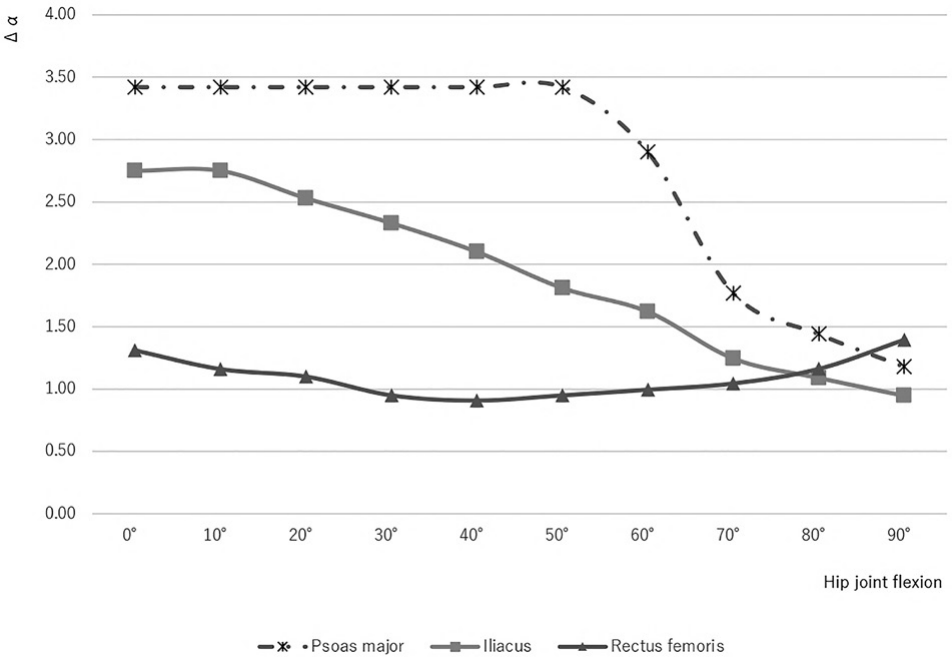
The relative rotation speed is represented by the change in flexion angle due to a 1% shortening of the muscle fiber. The change of flexion angle (Δα) of the psoas major remained constant in mild hip joint flexions up to 50° and decreased steeply up to 90°. Δα of the iliacus remained constant in hip joint flexions up to 10° and decreased gradually in flexions up to 90°. That of the rectus femoris decreased slightly in mild flexions up to 40° and thereafter increased slightly up to 90° to be at the same level as 0°.

In the hip extension position (0°), the relative rotation speeds of the PM and IL were 2.6- and 2.1-times swifter than that of the rectus femoris, respectively. The relative rotation speed of the PM remained constant in mild hip joint flexions up to 50°, and thereafter in deep flexions, decreased steeply up to 90°. The relative rotation speed of the IL remained constant in hip joint flexions up to 10º and decreased gradually in flexions up to 90°. Conversely, the relative rotation speed of the rectus femoris decreased slightly in mild flexions up to 40° and became approximately 90% compared with that at 0°; thereafter, it increased slightly up to 90° to become the same level as that at 0° (Figure 5).

## Discussion

In this study, we evaluated the different action of the iliopsoas and the rectus femoris on the hip flexion by estimating their relative contribution to the maximal hip flexion torque and relative rotation speed, and found that the iliopsoas served as a rapid flexor, whereas the rectus femoris was a powerful flexor.

The joint torque or force is produced by several agonist muscles and determined by various factors including neural activities^34)^, muscle fiber types^35)^, and contraction speed^36)^. For improving performance in athletic sports, it is important to understand the contribution of individual agonist muscles in producing torque in the hip joint. However, to the best of our knowledge, this has not been investigated properly to date. Hip joint flexion is exerted mainly by the iliopsoas and rectus femoris, and other accompanying muscles including the sartorius and adductor longus. The importance of the iliopsoas and rectus femoris as hip joint flexors is indicated by their larger PCSA (9.9 + 7.7 + 13.5 = 31.1 cm^2^) than that of the sartorius and adductor longus (1.9 + 6.5 = 8.4 cm^2^)^26)^. The sartorius and adductor longus have much smaller PCSA values, and their force vectors deviate from the sagittal plane. Actually, Inai et al^37)^. considered the iliopsoas and rectus femoris as the hip flexors in a computer simulation study investigating the dynamics of hip flexors during sit-to-stand movements. A study on iliopsoas function reported that the iliopsoas was activated during increased hip flexion when the subjects moved from the standing position (0°; a fully extended position of the hip and knee joints) to one-leg flexion of the hip joint (90°)^18)^. Furthermore, the contribution ratio of the four hip flexors (the iliopsoas, rectus femoris, tensor fasciae latae, and sartorius) to the flexion torque changes depending on the posture during the measurement of activities in these muscles^9)^. However, the contribution ratios of the muscles to the flexion torque were not clarified in these studies. The relative contribution of the individual flexors to the joint torque could not be experimentally obtained either by measurement of joint torque or by EMG of the relevant muscles. The relative contribution of the individual flexors to the maximal joint torque could be estimated on a theoretical basis by calculating the indicator of maximal joint torque as the product of the PCSA and the MAL for the individual muscles. It is well known that the joint torque exerted by a muscle is the product of muscle tension and the MAL and that the PCSA is an index of the maximal exertion tension of the muscle^38)^. To date, the PCSA and MAL have been measured separately in different studies. In this study, we measured both of them with cadaveric dissection for the first time, thus providing novel findings. The PCSA was calculated from morphometric parameters, such as the muscle volume, FL, and pennation angle, which could be accurately measured in the isolated muscle specimens^26, 30)^^-^^32)^.

The PCSA of a given muscle varied between the present and previous studies, especially because of the large individual variation in the muscle mass. In the present study, we calculated the ratio of the PCSA among the three muscles and its coefficient of variation (CV; standard deviation divided by the average) and found that the CV for the ratio of PCSA was quite small in contrast to the large CV for the PCSA, supporting the reliability of the estimation of the relative contribution of each muscle to the maximal flexion torque (Table 2).

**Table 2:** The PCSA of the psoas major, iliopsoas, and rectus femoris, and its ratio to the total amount

	PCSA		Ratio of PCSA	
	Average ± standard deviations(cm^2^)	CV	Average ± standard deviations(%)	CV
Psoas major	5.45±2.85	0.52	23.58±4.45	0.19
Iliacus	6.91±2.98	0.43	30.74±5.88	0.19
Rectus femoris	10.88±6.00	0.55	45.68±8.10	0.18

PCSA, physiological cross-sectional area; CV, standard deviation divided by average. The PCSA exhibited a significant individual variation, which is represented by the large value of the CV. However, the ratio of the PCSA was almost stable among individuals, which is indicated by the small value of the CV.

The MAL of the iliopsoas and rectus femoris and its change under different hip joint angles have been calculated^39)^ using a method based on an interactive musculoskeletal modeling software and the lower limb model of Delp et al^40)^. Their results based on model simulation were in good agreement with the results found for the skeletal specimens in the present study. The small and stable value for the iliopsoas in mild flexions up to 60° could be explained by the bend of the insertion tendon on the femoral head, and the steep increase in deep flexions could be the result of its distance from the head. The MAL of the rectus femoris was maximal at a hip flexion of 40°, where the two vectors from the origin (anterior inferior iliac spine) to the center of the hip joint and the insertion (upper edge of the patella) crossed vertically.

The present study revealed the main agonists of hip joint flexion as two functionally important parameters for the first time: the relative contribution to the maximal flexion torque and the relative rotation speed by muscle contraction. From these parameters, different functional properties of the iliopsoas and rectus femoris became apparent.

We also showed that the rectus femoris was a more powerful flexor than the iliopsoas in mild hip flexions up to 60° and a less powerful flexor in deep hip flexions, which is in agreement with the larger activities of the iliopsoas in deep hip flexions^18)^. Regarding the relative contribution of the iliopsoas and rectus femoris to the maximal flexion torque, the rectus femoris plays a more important role in the shallow flexion of the hip joint during walking among the activities of daily living.

Conversely, it was also shown that the relative rotation speed by muscle contraction for the PM and IL was 3.6 and 1.9 times larger than that of the rectus femoris in mild hip flexions up to 50°, respectively. Regarding estimation of the rotation speed by contraction of the flexors, it was clarified that the relative rotation speed of flexion differs between the iliopsoas and rectus femoris muscles under the specific condition that each muscle has the same muscle contraction speed. It would be reasonable to argue that the iliopsoas is particularly important for sprinters, contributing significantly to leg swing speed. In addition, weak hip flection muscles pose a risk for falling, and the main cause of injury in people with weak hip flexion muscles is stumbling or falling while climbing stairs. Strengthening the iliopsoas makes it possible to lift the leg instantly, thereby preventing falls when climbing stairs or stepping over obstacles.

This study has some limitations. We did not measure the sarcomere length in each muscle; therefore, the FL for calculating the PCSA was not normalized by the ratio of the optimal and measured sarcomere length. Further refinements of the PCSA by normalizing the FL would be beneficial for achieving a more precise estimation of the relative contribution to the maximal torque and the relative rotation speed. In addition, muscle fibers include slow-twitch fibers (Type I), which have a slow contraction rate and are less likely to fatigue, and fast-twitch fibers (Type II), which have a high contraction rate and are prone to fatigue. In human muscle, slow- and fast-twitch fibers are present in different ratios. Lieber^24)^ indicated that the differences in muscle fiber types have little effect on exercise performance, although there are differences in the maximum contraction rates of Type I and Type II fibers. In the present study, the effect of different muscle fiber types on relative rotation speed was not considered. Further improvements that also consider the histological factors of the muscle fibers would be beneficial for achieving a more precise estimation of the relative contribution of each fiber type to the relative rotation speed.

In conclusion, the present study clarified the functional characteristics of the iliopsoas and rectus femoris based on the morphometric data from skeletal anatomical specimens and applied the results to the simple movement of hip flexion on a theoretical basis to provide a better understanding of the functional contribution of muscles experimentally to the actual movements of the hip joint. The results of the present study would provide useful information for the functional training of athletes and rehabilitation programs in the clinic. Future developments of this research technique would clarify the functional contribution of the individual muscles in complex joint movements.

## Funding

The authors received no financial support for the research.

## Author contributions

All authors have made substantial contributions to the manuscript. The details are as follows:

TK: Conceptualization, Methodology, Formal analysis, Investigation, Data curation, Visualization, Project administration, Writing - original draft, Writing - review & editing

TT: Resources, Writing - review & editing, Supervision

TN: Resources, Writing - review & editing, Supervision

TS: Conceptualization, Methodology, Data curation, Visualization, Writing - review & editing, Supervision

All authors approved the final version of the manuscript to be submitted.

## Conflicts of interest statement

The authors declare that there are no conflicts of interest.
